# Oncolytic therapy of a recombinant Newcastle disease virus D90 strain for lung cancer

**DOI:** 10.1186/1743-422X-11-84

**Published:** 2014-05-12

**Authors:** Zheng Chai, Peiyi Zhang, Fang Fu, Xueyun Zhang, Ying Liu, Lihua Hu, Xi Li

**Affiliations:** 1 State Key Laboratory of Veterinary Biotechnology, Harbin Veterinary Research Institute, Chinese Academy of Agricultural Sciences, 427 Maduan St., Nangang District, Harbin 150001, China; 2 Gastrointestinal Department of Heilongjiang Province Hospital, Harbin 150000, China

**Keywords:** Newcastle disease virus, Lung cancer, Oncolytic therapy, Recombinant virus, Nude mice

## Abstract

**Background:**

Lung cancer is one of the leading causes of deaths from cancer worldwide. Tumor virotherapy using naturally oncolytic Newcastle disease virus (NDV) has been shown to be safe and effective in preclinical studies and clinical trials. Previously, we have reported the NDV D90 strain that was isolated from natural source has an antiproliferative effect in human lung cancer cell line A549.

**Methods and results:**

In this study, we constructed a reverse genetics system based on the oncolytic NDV D90 strain and generated a recombinant NDV carrying a gene encoding enhanced green fluorescent protein (rNDV-GFP). The rescued virus rNDV-D90 and rNDV-GFP showed the similar characteristics of replication and apoptotic ability in lung cancer A549 cells, which suggested that the recombinant viruses sustained the property of tumor-selective replication and induced apoptosis of tumor cells. The athymic mice bearing implanted lung cancer were treated with the parental D90 virus, the rescued rNDV-D90 and rNDV-GFP *via* intratumoral injections, respectively. The results showed that the recombinant viruses as well as the parental D90 virus significantly suppressed the loss of body weight and tumor growth.

**Conclusions:**

The study provides a new platform to develop effective therapeutic agents for tumor treatment. The availability of the reverse genetics system for NDV D90 strain will make it possible to develop novel recombinant oncolytic viruses based on the NDV D90 strain for improving the efficacy of tumor treatment.

## Background

Lung cancer was the most common cause of death from cancer worldwide [1]. Non-small cell lung cancer (NSCLC) accounts for approximately 80% of all cases of lung cancer. The most common types of NSCLC are adenocarcinoma, squamous and large cell [2]. Due to the lack of early diagnostic methods, many patients usually lose the opportunity of surgical treatment [3]. Till lately there were no consensus guidelines for the management of NSCLC. The overall survival and quality of life in patients with advanced NSCLC remains highly unsatisfactory despite the chemotherapy and radiotherapy could provide many options.

Oncolytic Newcastle disease viruses (NDV) can selectively replicate and destroy tumor cells. The inherent oncolytic property of NDV has been reported to derive from defective Interferon signaling pathways in tumor cells [4], while normal cells with an effective antiviral response hamper viral replication, which provides a mechanism for using NDV as a safe and effective tumor therapeutic vaccine [5]. The oncolytic NDV have been used clinically as a promising experimental therapy for over 50 years. To date, three strains of NDV, MTH-68, NDV-HUJ and PV701 are being used in phase I/II clinical trials for tumor treatment, suggesting that NDV is a safe and effective therapeutic agent for cancer therapy [6-9].

NDV, also known as avian paramyxovirus serotype 1, is a nonsegmented, negative-strand RNA virus of the family Paramyxoviridae with a natural avian host range. The genome of NDV is approximately 15 kb in length that encodes 6 structural proteins, nucleoprotein (NP), phosphoprotein (P), matrix protein (M), fusion protein (F), hemagglutinin-neuraminidase (HN) and the large protein (L) [10]. Based on the pathogenicity for avian species, NDV isolates are categorized into velogenic, mesogenic or lentogenic. The virulence of NDV is primarily determined by the F0 cleavage site. The F proteins of mesogenic and velogenic strains typically contain a polybasic cleavage site ((R/K)RQ(R/K)R↓F) [11], whereas lentogenic NDV strains characteristically have one basic residue at the -1 and -4 positions in the cleavage site (G/E)(K/R)Q(G/E)R↓L) [12,13].

In previous experiments, we have shown that the NDV D90 strain isolated from natural sources could induce apoptosis of human lung adenocarcinoma cell line A549 *via* a caspase-dependent pathway, significantly inhibit the expression of Bcl2 protein and show anti-proliferative effect but had no significant effect on normal cells *in vitro*[14]. However, there was no report for therapeutic effect of NDV D90 strain *in vivo*. In this study, we developed a reverse genetics system based on the NDV D90 strain and generated a recombinant NDV strain expressing enhanced green fluorescent protein (EGFP). Furthermore, we investigated the characteristics of the recombinant NDV D90 strains, including replication ability and anti-tumor activity *in vitro* and *in vivo*. Our results showed that both the rescued NDV D90 strain (rNDV-D90) and rNDV-GFP had tumor therapeutic efficacy *in vitro* and *in vivo*. The reverse genetic system for NDV D90 strain could provide a platform to develop a gene delivery system for tumor virotherapy, and afford the opportunity to develop improved therapeutic vectors engineered for increased antitumor efficacy.

## Results

### Construction of a reverse genetics system for NDV D90 strain

**Figure 1:**
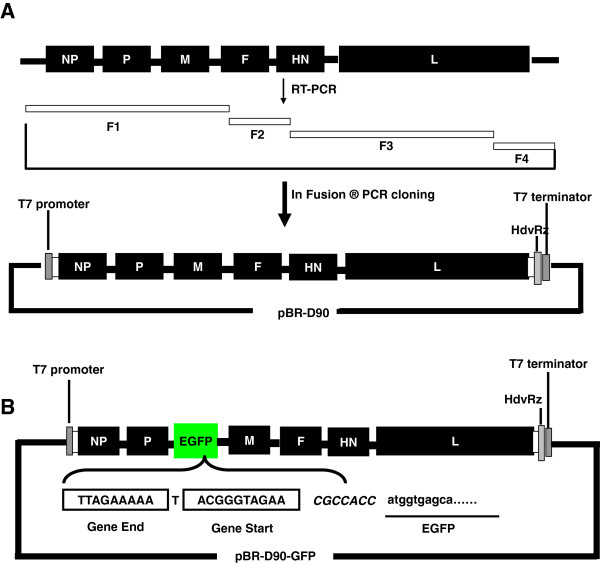
**Schematic diagram of NDV-D90 and recombinant NDV-GFP cDNA clone construction. (A)** Four cDNA fragments, marked by F1, F2, F3 and F4, were generated by RT-PCR from NDV D90 genomic RNA. These cDNA fragments were sequentially cloned and assembled into a modified pBR322 vector using In-Fusion^®^ PCR cloning kit (Clontech, USA). **(B)** The open reading frame of the EGFP gene was cloned into the intergenic region between the P and M genes in the D90 full-length clone as an additional transcription unit. The NDV Gene Start and Gene End signal sequences and the EGFP sequences are boxed or underlined.

The complete viral genome of D90 were cloned and sequenced to determine the complete nucleotide sequence of NDV D90 strain. The result showed that the complete genome length of D90 strain is 15,192 nt. The NDV D90 reverse genetics system contains a genome plasmid of the complete antigenomic cDNA and the helper plasmids. All of the plasmids were constructed as described in Methods. The full-length cDNA clone was constructed through four steps of RT-PCR and In-Fusion PCR cloning (Figure 1A).

**Figure 2:**
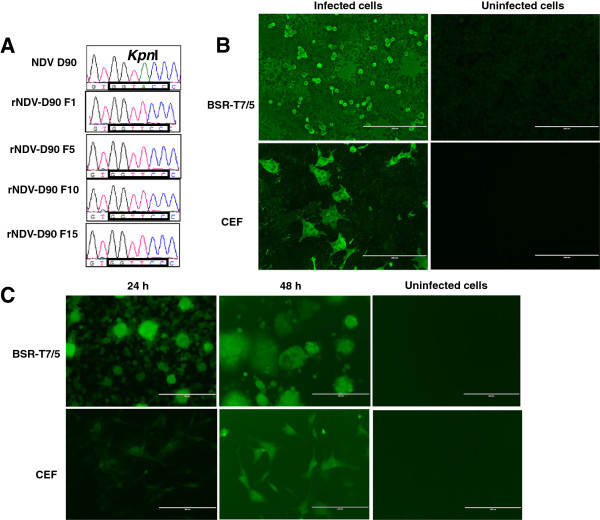
**Identification of the recovered Newcastle disease viruses. (A)** The genetic markers of the rescued NDV-D90 were confirmed by sequencing. The *Kpn*I restriction enzyme site in the L gene was eliminated by site-directed mutagenesis (A13996T) as the genetic marker. **(B)** The rescued rNDV-D90 infected BSR-T7/5 or CEF cells at an MOI of 0.01. After 48 h, the expressions of viral proteins were confirmed by IFA. Uninfected cells served as the negative control. **(C)** EGFP expression in rNDV-GFP-infected BSR-T7/5 or CEF cells. BST-T7/5 or CEF cells were infected with rNDV-GFP at a MOI of 0.01. Cells were observed at 24 h and 48 h post-infection.

To rescue recombinant NDV D90 (rNDV-D90), the plasmid pBR-D90 and three helper plasmids were co-transfected into BSR-T7/5 cells. After 3 days, the transfection supernatant was inoculated into 9- to 11-day-old embryonated SPF chicken eggs. The allantoic fluid (AF) was then harvested 3 days after inoculation and showed strong hemagglutinating (HA) activity. The presence of the genetic marker was confirmed by sequence analysis (Figure 2A). The rescued rNDV-D90 was further verified by Immunofluorescence assay (IFA) after infection of BSR-T7/5 and chicken embryo fibroblast (CEF) cells, respectively. As shown in Figure 2B, specific fluorescence was observed in the BST-T7/5 or CEF cells infected with rNDV-D90, but not in uninfected cells. These results demonstrated that rescued rNDV-D90 was generated successfully using the reverse genetics system.

### Generation of recombinant NDV carrying EGFP gene

The enhanced green fluorence protein (EGFP) gene ORF was inserted into the P-M gene junction region, resulting in the plasmid pBR-D90-GFP (Figure 1B). The plasmid was constructed as described in Methods and verified by DNA sequencing. To generate recombinant virus, pBR-D90-GFP was co-transfected with the three helper plasmids into BSR-T7/5 cells. The rescued rNDV-GFP viruses were collected. To examine expression of the EGFP gene, BST-T7/5 and CEF cells were infected with rNDV-GFP. At 24 h and 48 h post-infection, EGFP expression was observed in the rNDV-GFP-infected cells, indicating that the recombinant virus rNDV-GFP was generated (Figure 2C).

### Replication characteristics of recombinant NDV

**Figure 3:**
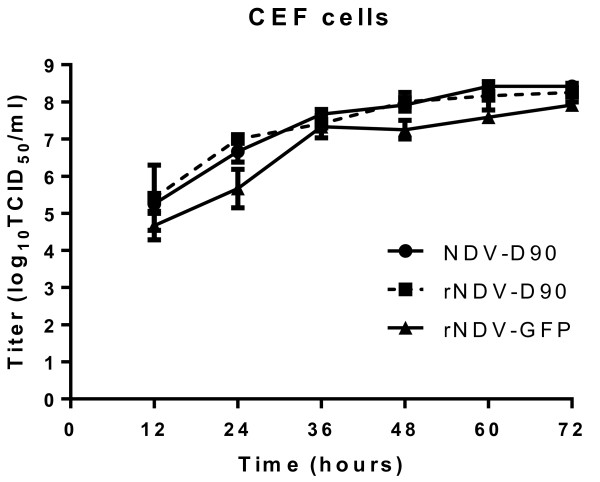
**Growth dynamics of the recombinant viruses in CEF cells.** The CEF cells were infected with rNDV-D90, rNDV-GFP or D90 strain at a MOI of 0.01. Every 12 h post-infection, the cells were harvested. Virus titers at each time point were determined by TCID_50_ titration in CEF cells.

To evaluate the growth characteristics of the rescued recombinant viruses in the CEF cells, viral titers of the supernatants that were collected from infected CEF cells at 12 h intervals for a period of 72 h were determined by TCID_50_. As shown in Figure 3, the growth properties of rNDV-D90 or rNDV-GFP in the CEF cells were similar to those of the parental virus NDV-D90. The result demonstrated that the recombinant viruses had mostly retained the growth characteristics of the parental virus in the CEF cells.

### Oncolytic ability of the recombinant viruses in lung cancer A549 cells

**Figure 4:**
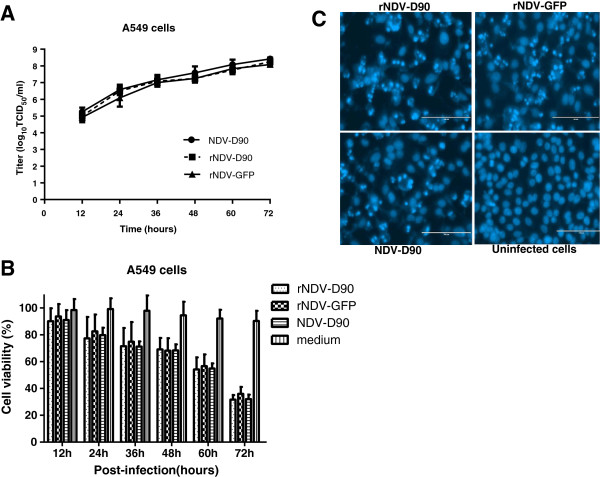
**Characteristics of recombinant NDV in lung cancer cell line A549. (A)** Growth dynamics of the recombinant viruses in A549 cells. The A549 cells were infected with rNDV-D90, rNDV-GFP or D90 strain at a MOI of 0.01. Every 12 h post-infection, the cells were harvested. Virus titers at each time point were determined by TCID_50_ titration in A549 cells. **(B)** Effect of rNDV-D90 or rNDV-GFP on cell viability was assessed in A549 cells by MTT assay. Data are presented as means (±SD) derived from six independent measurements. **(C)** DAPI staining. A549 cells were infected with rNDV-D90 or rNDV-GFP for 16 h at a MOI of 0.1. Nuclei are stained blue. The cells infected with viruses show the characteristics by chromatin condensation with brighter fluorescence.

The growth kinetics of the recombinant viruses and the parental NDV D90 viruses were investigate in A549 cells. As shown in Figure 4A, all tested recued viruses replicated with the similar kinetics as the parental virus in lung cancer cells.

To evaluate the oncolytic ability of rNDV-D90 or rNDV-GFP, the lung cancer A549 cells were infected with recombinant viruses at an MOI of 0.01, and the cell viability were assayed at various time points after infection. The parental NDV-D90 was a control. As shown in Figure 4B, the survival rates of the A549 cells decreased significantly by viral infection from 24 h post-infection and were lower 40% at 72 h post-infection. Furthermore, there was no significant difference in the cell viability of the lung cancer cells infected with rNDV-D90, rNDV-GFP or NDV-D90 during the whole experiment (*P* > 0.05).

To examine the ability of rNDV-D90 or rNDV-GFP to induce apoptosis in A549 cells, the infected cells with recombinant viruses were visualized by DAPI staining. As shown in Figure 4C, the lung cancer cells with recombinant viruses infected showed chromatin condensation, indicating that the rescued viruses caused apoptosis of A549 cells. These results indicate that both rNDV-D90 and rNDV-GFP still retain the intrinsic oncolytic properties as well as the parental NDV-D90.

### Recombinant viruses inhibit tumor growth in treated mice

**Figure 5:**
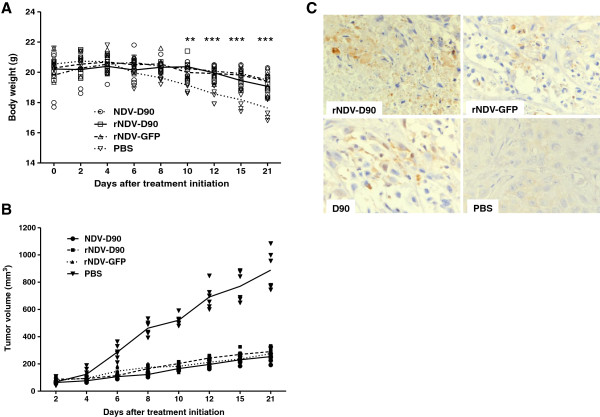
**Treatment of A549 tumor-bearing nude mice with recombinant viruses.** Female athymic mice bearing A549 cell tumors were treated with the parental D90, rNDV-D90 or rNDV-GFP (10^7^ TCID_50_/mouse) by intratumoral injections. PBS-treated mice were taken as controls. **(A)** Body weights were monitored. **P < 0.01, ***P < 0.001. **(B)** Tumor volume was measured and recorded. **(C)** Immunohistochemical staining of cleaved caspase-3 in tumor sections from the tumor-bearing mice.

To assess the therapeutic potential of rNDV-D90 and rNDV-GFP viruses *in vivo*, tumor xenografts were established using lung cancer A549 cells. Mice were injected subcutaneously with lung cancer A549 cells in the right flank to develop primary tumor. After 20 days, the tumor-bearing mice were treated with a total of four intratumoral injections of the parental NDV-D90, rNDV-D90, rNDV-GFP or PBS. As shown in Figure 5A, treatment with rNDV-D90, rNDV-GFP or NDV-D90 suppressed the loss of body weight compared with the PBS control group. Furthermore, there were significant differences in body weight of mice among the viral treatment groups and PBS group (Figure 5A). As shown in Figure 5B, the tumor volume of the mice treated with NDV-D90, rNDV-D90 or rNDV-GFP was much smaller than that of PBS group after four treatments with viruses (*P* < 0.01). There was about a 70% decrease in tumor volume in mice treated with rNDV-D90 or rNDV-GFP compared with PBS control at 21 days after the initial treatment (Figure 5B). Furthermore, these results (Figure 5A and B) indicated that there was no difference in tumor growth inhibition between rNDV-D90 and the recombinant virus with exogenous gene (*P* < 0.01).

We next investigated whether recombinant viruses would inhibit the tumor growth *via* a caspase-3 pathway in mice. The histological test of tumor tissue treated with parental NDV or recombinant viruses revealed an occurrence of apoptosis as shown in Figure 5C, indicating that the oncolytic efficacy of recombinant viruses depended on the apoptosis of tumor cells *in vivo*.

## Discussion

NSCLC is one of the leading causes of deaths from cancer worldwide, therefore, improvements in diagnostics and treatments are urgently needed [15]. Oncolytic viruses have the potential to break immunological tumor tolerance, and generate antitumor immunity. Therefore, oncolytic viruses represent a promising novel immunotherapy strategy, which may be optimally combined with existing therapeutic modalities [16]. In this study, a rescued virus rNDV-D90 by reverse genetics system based on the complete sequence of oncolytic NDV-D90 strain that we isolated from natural source was generated. Furthermore, we showed that rNDV-D90 could stably carry and express an EGFP gene positioned between the P and M gene of NDV. Importantly, the rNDV-D90 and rNDV-GFP showed the similar growth and oncolytic characteristics in lung cancer cell line A549 and tumor growth inhibition in nude mice as well as the parental NDV-D90 virus.

Naturally occurring oncolytic viruses include the double-stranded RNA reovirus and single-stranded RNA NDV and vesicular stomatitis virus, while human DNA viruses, including adenoviruses, vaccinia virus and herpes simplex viruses (HSV) have been genetically modified in a variety of ways to provide tumor selectivity [16]. Due to significant virulence associated with the use of some human pathogens, animal viruses were explored as a promising therapeutic agent for tumor. Replication of NDV seems to be restricted in other species, including human cells [4]. Unlike HSV-1 or adenovirus, NDV does not have significant adverse effects in human, suggesting that NDV is attractive as a potential therapeutic vaccine [17]. Although oncolytic virotherapy has developed rapidly, the oncolytic properties of these viruses need improvement to achieve maximal oncolytic effect.

Reverse genetics has been explored as an approach to improve the oncolytic activity of NDV [18]. For the purposes of studying the oncolytic mechanism and enhancing the oncolytic effect, we attempted to establish the reverse genetics system based on NDV D90 strain. As the same as the parental D90 strain, the rNDV-D90 strain was a mesogenic strain (the mean death time of embryos was 86.61 h) that contains a polybasic cleavage site ((R/K)RQ(R/K)R↓F) (Data was not shown). It was reported that the replication of recombinant NDV was delayed by insertion of a foreign gene, which was inserted in the gene junction [19]. We here introduced a foreign gene in the noncoding region between the P and M genes to generate the recombinant virus rNDV-GFP. The rNDV-GFP virus expressed high levels of exogenous protein (Figure 2C) and showed the similar replication characteristics as well as the parental virus and rNDV-D90 in CEF cells (Figure 3), which demonstrated that the noncoding region between the P and M genes could be a suitable position for NDV D90 strain to carry foreign genes and provides the basis to design recombinant vector vaccines.

Various reports have shown that anti-tumor mechanism induced by NDV is dependent on induction of apoptosis [14,20-22]. In the lung cancer cell line A549, the rNDV-D90 and rNDV-GFP viruses showed the similar replication ability as well as the parental D90 virus *via* growth curve (Figure 4A). The results of MTT assay (Figure 4B) and DAPI-staining analysis (Figure 4C) demonstrated that both the rescued NDV and recombinant virus with the insertion of exogenous gene had strong oncolytic effect in lung cancer cells. These data suggested that the recombinant viruses retained the oncolytic characteristics in lung cancer cells *via* apoptosis.

The early reports have shown that oncolytic NDV strains are capable to induce tumor cell apoptosis *in vivo*[5,23,24]. However, some recombinant NDV that induced apoptosis in tumor cells did not show significant differences in tumor size, body weight between the treated mice and the control mice [25]. Furthermore, the tumor therapy *in vivo* was not progressed using the parental D90 virus in previous study. In this study, we investigated whether the parental and recombinant viruses could be effective therapeutic agents to suppress tumor growth in NSCLC model of nude mice. In the whole experiment, the body weights of mice that were treated with viruses NDV-D90, rNDV-D90 or rNDV-GFP did not significantly change, suggesting that recombinant viruses are sufficient safe not to cause side effect. As shown in Figure 5B, rNDV-D90 and rNDV-GFP as well as the parental D90 virus resulted in significant inhibition of tumor growth compared with PBS control. Furthermore, there was no difference in anti-tumor efficacy among the parental NDV D90, rNDV-D90 and rNDV-GFP (*P* > 0.05), suggesting that the insertion of foreign gene could not reduce the oncolytic efficacy of NDV *in vivo*. In addition, NDV induces apoptosis by both intrinsic and extrinsic caspase-dependent pathways of apoptosis [22]. The intrinsic and extrinsic pathways both end at the point of the execution phase as a final pathway of apoptosis [26]. The pathway is initiated by the cleavage of caspase-3 and results in DNA fragmentation, degradation of cytoskeletal and nuclear proteins [26]. In this study, we performed an IHC study of apoptosis in nude mice with lung cancer A549 tumors xenografted. The lung cancer cells undergoing apoptosis were observed by IHC using an antibody against the active form of caspase-3, suggesting that NDV inhibits tumor growth *via* caspase-dependent apoptotic pathway *in vivo*. Despite complete regression of these tumors was not observed in tumor-bearing mice treated with the NDV D90 or recombinant viruses, the oncolytic properties of rNDV-GFP provide the basis to design recombinant vector for enhancing therapeutic efficacy by further reverse genetic modifications of NDV. Additional modification to rNDV-D90 will allow for the possibility of generating more effective agents compared with rNDV-D90.

## Conclusions

In summary, we established a reverse genetics system based on the oncolytic NDV D90 strain, and generated a recombinant NDV expressing the EGFP. Our results demonstrated that the rNDV-D90 and rNDV-GFP had similar oncolytic effect in tumor cells. Furthermore, the recombinant viruses as well as the parental NDV D90 showed the significant therapeutic efficiency in A549 tumor-bearing mice. Therefore, this study not only provides a novel therapeutic agent for human lung cancer but also generates a potential oncolytic viral vector for enhancing immunotherapeutic effect on tumors.

## Methods

### Ethics statement

This study was carried out in strict accordance with animal ethics guidelines and approved protocols. All animal studies were approved by the Animal Ethics Committee of Harbin Veterinary Research Institute of the Chinese Academy of Agricultural Sciences (SYXK (H) 2006–032).

### Cells and virus

BSR-T7/5 cells stably expressing the phage T7 RNA polymerase [27], chicken embryo fibroblast (CEF) cells and the human lung adenocarcinoma A549 cells were cultured in Dulbecco’s modified Eagle’s medium (Invitrogen, USA) supplemented with 10% fetal calf serum at 37°C in a humidified 5% CO2 incubator. The NDV D90 strain was prepared as reported previously [14].

### Construction of recombinant plasmids

Viral RNA was extracted using the TRIzol-LS reagent (Invitrogen, USA) according to the manufacturer’s instructions. The RT reaction was performed as the previous described [28]. The complete genomic sequence of the D90 strain was obtained by sequencing (Beijing Genomic Institute, China) using twelve primer pairs, which are available upon request.

To construct the helper plasmids, the open reading frames (ORFs) of NP, P, and L were amplified from the NDV-D90 cDNA using specific primers including Kozak consensus sequences (Table 1). The NP and P genes were cloned between the *EcoR*I and *Xba*I; the L gene between *Sal*I and *Not*I (Table 1 primers). The plasmids pCI-NP, pCI-P, and pCI-L were generated and confirmed by sequencing, respectively.

**Table 1:** Primer sequences used in the study

**Primer**	**Primer sequence (5′-3′)**^**a**^
D90-F4-F	ATAAACTAGT**ACGCGT**GGTtTCAGGCTTATATGCAG
D90-F4-R	ATGCCATGCCGACCCACCAAACAGAGATTTGGTGAATG
D90-F1-F	AATACGACTCACTATAGGACCAAACAGAGAATCTGTGAG
D90-F1-R	ACGCGTACTAGT**TTATAA**AGTGCCTGGATGGTCAGCTG
D90-F2-F	CATCCAGGCACT**TTATAA**TTTAGCTGGTGGCAATATG
D90-F2-R	TAATACGCGT**ACTAGT**AAGGGAACGATCCTAAATTC
D90-F3-F	TCGTTCCCTT**ACTAGT**TGAGATCCTCAAGGATGATAG
D90-F3-R	GCCTGAAACCACGCGTCGAGTGCAAGAGACTAACAATC
EGFP-F	GCGTCACACGGAATCCCGCGGAGTTAGAAAAAATACGGGTAGAACGCCACCatggtgagcaagggcgaggagctg
EGFP-R	CGCGAGGGGGGGCCCCCGCGGttacttgtacagctcgtccatgccg
NP-F-Kozak	TATT**GAATTC**GCCACCATGTCGTCTGTTTTCGACGAATAC
NP-R	TATA**TCTAGA**TCAGTACCCCCAGTCAGTGTC
P-F-Kozak	TATT**GAATTC**GCCACCATGGCCACTTTTACAGATGC
P-R	TATA**TCTAGA**TCAACCATTtAGCGCAAGGCG
L-F-Kozak	TATT**GTCGAC**GCCACCATGGCGGGCTCCGGTCCCGAAAG
L-R	TATA**GCGGCCGC**TTAAGAGTCATTATTACTGTAATATC

^a^The restriction enzyme sites within the primers were marked in bold type. Nucleotides shown in lower case letters represent the sequence from EGFP.

The plasmid pBR-THT, derived from pBR322 vector (Takara, China) containing the sequences of the T7 promoter, hepatitis delta ribozyme (HdvRz), and T7 termination signal was constructed. To construct the full-length genomic cDNA plasmid, a 90-nucleotide linker containing multiple restriction enzyme sites was introduced into plasmid pBR-THT-linker, using an In-Fusion PCR cloning kit (Clontech, USA). Four cDNA fragments, F1, F2, F3 and F4, coding for the complete sequence of the D90 virus genomic RNA, were generated by RT-PCR. These fragments were sequentially cloned into the plasmid pBR-THT-linker using an In-Fusion PCR cloning kit (Clontech, USA) (Figure 1A). Primer sets used to amplify the cDNA fragments were shown in Table 1. The *Kpn*I restriction enzyme site in the L gene was eliminated through one nucleotide mutation (A13996T) to serve as a genetic marker. The resulting full-length cDNA plasmid was designated pBR-D90.

To construct a full-length plasmid containing the EGFP gene, the full-length D90 cDNA clone pBR-D90 was used as a backbone to construct a recombinant cDNA clone containing the EGFP gene between the P and M genes as an additional transcription unit. A unique *Sac*II restriction enzyme site was introduced into the region between the P and M genes of the pBR-D90 infectious clone by overlap PCR. The EGFP gene was amplified using the primer pairs (Table 1) from pEGFP-N1 (Clontech, USA). The ORF of EGFP gene was engineered to contain the NDV gene-start and gene-end signal sequences and inserted into the *Sac*II restriction enzyme site in pBR-D90 adhering to the “rule of six” (Figure 1B) [29,30]. The positive clone was confirmed by sequencing and designated as pBR-D90-GFP.

### Rescue of the recombinant viruses

To rescue the recombinant virus rNDV-D90 and rNDV-GFP, BSR-T7/5 cells were cultured to 70% confluence in a six-well plate and transfected with total 8 μg mixtures of the indicated plasmids including pBR-D90 or pBR-D90-GFP, pCI-NP, pCI-P, and pCI-L at the ratio of 4:2:1:1 using LipofectamineTM 2000 (Invitrogen, USA) according to the manufacturer’s instructions. At 6 h post-transfection, the cells were washed three times with phosphate buffered saline (PBS) and cultured in opti-MEM medium (Invitrogen, USA). After 3 days, the culture supernatant was harvested and inoculated into the allantoic cavities of 9- to 11-day-old embryonated SPF eggs until the AF showed NDV-specific HA activity. The rescued rNDV-D90 was serially passaged 15 times in 9- to 11-day-old embryonated SPF eggs.

To identify recombinant NDV, the 5 th, 10 th and 15 th passages of the AF containing rNDV-D90 viruses were collected. Viral RNA from the rNDV-D90-infected chicken embryo AF was extracted and the RT reaction was performed. Primers 10-F and 10-R (Table 1) were used to amplify the part coding region of L protein where contains the genetic marker of rNDV-D90. The PCR products were confirmed the presence of the artificially introduced genetic markers by DNA sequencing.

### Immunofluorescence assay

BSR-T7/5 cells or CEF cells were cultured in a 96-well plate and infected with rNDV-D90 at a multiplicity of infection (MOI) of 0.01. At 48 h post-infection, the cells were fixed with absolute ethyl alcohol, and then incubated using anti-chicken NDV polyclonal sera (1: 40 dilution) and the FITC-conjugated goat anti-chicken IgG antibody (1: 200 dilution) (Sigma, USA). Uninfected cells were as the negative control. The samples were visualized with EVOS fl fluorescence microscope (AMG, USA).

### Live cell imaging

BSR-T7/5 cells or CEF cells were cultured in a 24-well plate and infected with rNDV-GFP at a MOI of 0.01. Cells were observed with EVOS fl fluorescence microscope (AMG, USA). Live cell imaging was recorded at 24 h and 48 h post-infection.

### Growth characteristics of the recombinant viruses

The growth characteristics of the recombinant virus were evaluated on CEF cells and A549 cells, respectively. Confluent monolayers of CEF or A549 cells were infected with rNDV-D90, rNDV-GFP or the parental D90 strain in 6-well plates at a MOI of 0.01. Every 12 h post-infection, the cellular monolayers of CEF or A549 cells were harvested by freeze-thawing three times. Virus titers were determined as 50% tissue culture-infective dose (TCID_50_) per 0.1 ml. Each assay was repeated three times.

### MTT assay

The viability of cells was assessed with a 3-(4,5-dimethylthiazol-2-yl)-2,5-diphenyltetrazolium bromide (MTT) cell proliferation and cytotoxicity assay kit (Keygentic, China) with six replicates for rNDV-D90, rNDV-GFP, NDV-D90 or medium. At 12, 24, 36, 48, 60, 72 h post-infection, cell viability was determined by incubating the cells with MTT. Absorbance at 490 nm was determined with ELISA microplate readers (BioTek Instruments, Winooski, VT).

### DAPI staining

A549 cells were infected with rNDV-D90, rNDV-GFP or NDV-D90 at a MOI of 0.01 for 24 h. Cells were stained with DAPI (Keygentic, China). DAPI staining was performed as described previously [31]. In brief, prior to staining, the cells were fixed with 4% paraformaldehyde for 30 min at room temperature. DAPI was added to the fixed cells for 30 min, and then examined by fluorescence microscopy. Apoptotic cells were identified by condensation and fragmentation of nuclei.

### Animal study

Six-week-old female athymic nude mice were purchase from Shanghai Slac Laboratory Animal Company and housed in a pathogen-free environment. All the mice were injected subcutaneously into the outer thigh with 5 × 10^6^ A549 cells per mice. Solid tumors became visible after 10 days. The tumor sizes were determined as the product of the perpendicular diameters of the tumors. Mice were randomly divided into four groups. Each group contained 6 mice. On day 20 post-inoculation, the mice were treated by intratumoral injection of 1 × 10^7^ TCID_50_ of NDV D90, rNDV-D90, rNDV-GFP or PBS in a total volume of 100 μl. The treatments were repeated every other day for a total of four treatments. Both tumor volume and body weight were measured at 2, 4, 6, 8, 10, 12, 15 and 21 day after the treatment initiation. All animals were euthanized 21 days after treatment was initiated. At that time, all animals reached the humane clinical endpoint for the study which was taken as the point when the tumor exceeded 10% of normal body weight or was greater than 1.5 cm diameter of the tumor.

### Immunohistochemistry

The tumors were fixed in 10% neutral buffered formalin solution. They were dehydrated, embedded in paraffin, and cut into 5 μm sections. The primary polyclonal rabbit anti-cleaved caspase-3 (Asp175) antibody (Cell signaling, USA) was diluted at 1:300. Subsequently, the sections were incubated with anti-rabbit secondary antibody (Sigma, USA) was added to the sections. Following a PBS wash, DAB substrate was performed. The sections were analyzed *via* microscopy.

### Statistical analysis

The statistical analysis was performed using two-way ANOVA analysis. *P* values less than 0.05 were considered statistically significant. Data were presented as mean values ± the standard deviation (SD).

## Competing interests

The authors declare that they have no competing interests.

## Authors’ contributions

ZC, PZ, FF and XL designed the experiments. ZC, XZ, LH and YL performed the experiments. ZC and XL analyzed the data and wrote the manuscript. All authors read and approved the final manuscript.
